# Inhibition of Prostaglandin Reductase 2, a Putative Oncogene Overexpressed in Human Pancreatic Adenocarcinoma, Induces Oxidative Stress-Mediated Cell Death Involving *xCT* and *CTH* Gene Expressions through 15-Keto-PGE_2_

**DOI:** 10.1371/journal.pone.0147390

**Published:** 2016-01-28

**Authors:** Emily Yun-Chia Chang, Yi-Cheng Chang, Chia-Tung Shun, Yu-Wen Tien, Shu-Huei Tsai, Siow-Wey Hee, Ing-Jung Chen, Lee-Ming Chuang

**Affiliations:** 1 Department of Internal Medicine, National Taiwan University Hospital, Taipei, Taiwan; 2 Graduate Institute of Medical Genomics and Proteomics, National Taiwan University, Taipei, Taiwan; 3 Institute of Biomedical Science, Academia Sinica, Taipei, Taiwan; 4 Center for Obesity, Life style and Metabolic Surgery, National Taiwan University Hospital, Taipei, Taiwan; 5 Department of Forensic Medicine and Pathology, National Taiwan University Hospital, Taipei, Taiwan; 6 Department of Surgery, National Taiwan University Hospital, College of Medicine, National Taiwan University, Taipei, Taiwan; 7 Terry Fox Laboratory, BC Cancer Agency, Vancouver, British Columbia, Canada; 8 Institute of Molecular Medicine, College of Medicine, National Taiwan University, Taipei, Taiwan; 9 Department of Medicine, College of Medicine, National Taiwan University, Taipei, Taiwan; University of South Alabama Mitchell Cancer Institute, UNITED STATES

## Abstract

Prostaglandin reductase 2 (*PTGR2*) is the enzyme that catalyzes 15-keto-PGE_2_, an endogenous PPARγ ligand, into 13,14-dihydro-15-keto-PGE_2_. Previously, we have reported a novel oncogenic role of *PTGR2* in gastric cancer, where *PTGR2* was discovered to modulate ROS-mediated cell death and tumor transformation. In the present study, we demonstrated the oncogenic potency of *PTGR2* in pancreatic cancer. First, we observed that the majority of the human pancreatic ductal adenocarcinoma tissues was stained positive for *PTGR2* expression but not in the adjacent normal parts. *In vitro* analyses showed that silencing of *PTGR2* expression enhanced ROS production, suppressed pancreatic cell proliferation, and promoted cell death through increasing 15-keto-PGE_2_. Mechanistically, silencing of *PTGR2* or addition of 15-keto-PGE_2_ suppressed the expressions of solute carrier family 7 member 11 (*xCT*) and cystathionine gamma-lyase (*CTH*), two important providers of intracellular cysteine for the generation of glutathione (GSH), which is widely accepted as the first-line antioxidative defense. The oxidative stress-mediated cell death after silencing of *PTGR2* or addition of 15-keto-PGE_2_ was further abolished after restoring intracellular GSH concentrations and cysteine supply by N-acetyl-_L_-cysteine and 2-Mercaptomethanol. Our data highlight the therapeutic potential of targeting *PTGR2*/15-keto-PGE_2_ for pancreatic cancer.

## Introduction

Pancreatic cancer is known for its poor prognosis due to its high resistance to standard chemotherapeutic treatment. Because it is still an obstacle detecting pancreatic cancer in its early stage, the majority of patients are diagnosed when the tumor has reached an inoperable stage [1–3]. Thus, it is important to develop novel strategies, new molecular targets, and more effective treatments to improve the prognosis for pancreatic cancer patients.

Prostaglandin reductase 2 (PTGR2) catalyzes the NADPH-dependent reduction of 15-keto-PGE_2_ into the downstream unstable metabolite 13,14-dihydro-15-keto-PGE_2_ [4, 5]. 15-keto-PGE_2_ is a well-established endogenous ligand of peroxisome proliferator-activated receptor γ (PPARγ), which is a master regulator of adipogenesis and lipid metabolism [4, 6]. Over-expression of PTGR2 suppressed while inactivation of PTGR2 increased PPARγ-mediated adipogenesis and lipid metabolism. Besides the regulation of adipogenesis and lipid metabolism, extensive research has also established the role of PPARγ in tumor progression and cancer metastasis [7–11]. PPARγ ligands, including synthetic ligands and prostaglandin metabolites, have repeatedly demonstrated to impact on cancer progression either independently or through PPARγ activation as well [12–17].

Previously, we also established a novel role of PTGR2 in cancer biology, where PTGR2 takes part in ROS-mediated cell death and tumor transformation in gastric cancer. We showed both *in vitro* and *in vivo* that knockdown of *PTGR2* suppressed tumor growth and induced apoptosis through ROS-mediated signaling involving ERK1/2 and caspase 3 activities. We further observed strong PTGR2 staining in tumor part relative to adjacent non-tumor areas in gastric tissues. Importantly, tumor-part PTGR2 stain intensity negatively correlated with the survival of patients with intestinal type gastric cancer [18]. Nonetheless, how PTGR2 affects ROS level still remains unknown.

Excess ROS is often detrimental to cells. However, ROS can also promote pro-oncogenic signaling pathways and aids in cancer progression. Thus, cancer cells often adapt to higher oxidative stress by carrying a higher antioxidant capacity to maintain ROS to levels advantaged to them without inducing cell death [19, 20]. Numerous studies in identifying novel therapeutic strategies for cancer have also shown that targeting the antioxidant signaling is effective in triggering cancer cell death [21–24]. Amongst all, glutathione (GSH) is widely known to serve as the first line antioxidative defense mechanism [25], and cystathionine gamma-lyase (CTH) and solute carrier family 7 member 11 (xCT) are two important providers of intracellular cysteine, the precursor for the generation of GSH.

CTH is the enzyme that catalyzes the hydrolysis of cystathionine to form cysteine, which can be further metabolized to form glutathione. Past studies have shown that *CTH*-deficient mice acquired cystathioninuria and were more sensitive to oxidative injury [26]. *CTH*-Knocked-down melanoma cells not only showed suppressed proliferation rates and induced H_2_O_2_ sensitivity, but also induced senescence [27]. A recent study further proved in RNAi experiments that CTH is essential for the maintenance of cellular GSH concentration as well as antioxidative defense [28]. xCT, the functional subunit of the system X_c_^-^ cystine/glutamate antiporter, is another important provider of cellular cysteine for the generation of GSH. Furthermore, xCT is often upregulated and implicated in cancer because not only is cysteine/cysteine uptake from the microenvironment crucial for cancer cell growth and viability, but also xCT helps modulate tumor microenvironment in general leading to growth advantage [29, 30]. As a result, ample evidence has shown the induced expression and the crucial role of xCT in numerous human carcinomas, including hepatocellular carcinoma, leukemia, and brain, prostate, ovarian, gastric, colon, and pancreatic cancers. Studies of xCT in these cancers all demonstrated that knocking down *xCT* or blocking its activity led to suppressed proliferation, induced ROS level and cell death, and tumor regression [31–39].

PTGR2 is found to be expressed in pancreatic cancer tissues, but absent in normal pancreatic tissues. Several studies have also documented the ability of PPARγ ligands to attenuate growth and increase cell death of pancreatic cancer cell lines [40–43]. In the present study, we provided evidence showing that the oncogenic property of PTGR2 is not only specific to gastric cancer, but also impact on pancreatic cancers. Importantly, we showed for the first time that the impact of PTGR2 on cancer cell death seemed to be the resultant of a defective antioxidative defense system involving xCT and CTH, both of which are important regulators of intracellular reduced GSH. Moreover, the impact of PTGR2 on oxidative stress-induced pancreatic cell death was associated with the changing concentration of 15-keto-PGE_2_, and seemed to involve both PPARγ-dependent and–independent pathways. These data suggest the potential of targeting PTGR2 and the redox status of cancer cells for future therapeutic purposes.

## Materials and Method

### Ethics Statement

The study was conducted according to the regulations of the Institutional Review Board of National Taiwan University Hospital (NTUH) and the specimens were anonymous and analyzed in a blinded manner. All pancreatic cancer tissue specimens are from the National Taiwan University Hospital, Taipei, Taiwan. All patients were given informed consent, which was approved by the Institutional Review Board of NTUH (201303029RINC), and every patient had submitted a written consent before operation. The Institutional Review Board of NTUH has specifically approved the specimens for use in this study and has specifically approved this study.

### Human Tissue Immunohistochemistry

76 patients with pancreatic ductal adenocarcinoma (PDAC) who received surgery and pathological assessment at the National Taiwan University Hospital (NTUH) were recruited for this study. This study was conducted according to regulations of the Institutional Review Board of NTUH and the specimens were anonymous and analyzed in a blinded manner. Immunohistochemistry was performed using the avidin-biotin complex immunoperoxidase method. Briefly, sections from formalin-fixed, paraffin-embedded tumor specimens were prepared, and immunohistochemical staining was performed using mouse monoclonal antibody against human PTGR2 or nonimmune IgG, and examined using a bright-field microscope. PTGR2 staining positivity was meticulously examined by one pathologist (Dr. Chia-Tung Shun) and classified into two groups: positive and negative for PTGR2 staining.

### Materials, Cell Culture and Transfection

Human pancreatic cancer cell lines PL45, MIA PaCa-2, PANC-1, BxPC-3 and Capan-2 (gifts from AbGenomics BV, Taiwan Branch, Neihu Taipei, Taiwan) were cultivated in Dulbecco’s modified Eagle’s medium (DMEM) supplemented with 10% fetal bovine serum (Gibco, Grand Island, NY), 2 mM glutamine, and 100 units/ml of penicillin and streptomycin. Cellular environment was maintained at 5% CO_2_ at 37°C incubator. For the RNA interference assay, cells were transfected with 100 nM of siRNA targeting PTGR2 (*si-PTGR2*) or non-targeting control siRNA (*si-Cont*) (Dharmacon, Lafayette, CO) using TurboFect^TM^ reagent (Fermentas) in accordance with the manufacturer’s protocol. The sequences of the siRNAs are as follows: *si-PTGR2*, 5′-CGAAUGGAAGAAGUCUAUUUAUU-3′; and *si-Cont*, 5′-UGGUUUACAUGUCGACUAAUUA-3′. For drug treatment assays, cells were treated with a final concentration of 1000X dilution of dimethyl sulfoxide (DMSO), 10 μM BRL, 10 μM GW9662, 5 μM N-acetyl-_L_-cysteine (NAC) (Sigma-Aldrich, St. Louis, MO), 55 μM 2-Mercaptomethanol (2-ME) (Gibco), and 20 μM 15-keto-PGE_2_ (Cayman Chemical, Ann Arbor, MI).

### Western Blot Analysis

Whole-cell lysates were prepared with RIPA buffer (BioVision, Mountain View, CA) containing protease and phosphatase inhibitor cocktail tablets (Roche Applied Science, Indianapolis, IN), and the protein concentration was determined by Bio-Rad Protein assay (Bio-Rad, Philadelphia, PA). Equivalent amounts of protein were resolved by SDS-PAGE and transferred to polyvinylidene fluoride (PVDF) microporous membrane (Millipore, Billerica, MA). Non-specific-antibody-binding sites were blocked with 5% skim milk in PBS containing 0.1% Tween 20 (PBST), and membranes were probed with the following antibodies: PTGR2 (gift from AbGenomics BV), Hsp70, CTH, and SLC7A11/xCT (GeneTex, Irvine, CA), 15-PDGH (Cayman Chemical), Catalase, GAPDH, COX1 and COX2 (Epitomics, Burlingame, CA), and PPARγ (Santa Cruz, Santa Cruz, CA). Secondary antibodies were conjugated to Horseradish Peroxidase or Alkaline Phosphatase (Santa Cruz), and peroxidase activity or phosphatase activity was visualized using Chemiluminescent HRP Substrate (Millipore) or BCIP/NBT Phosphatase Substrate (KPL, Gaithersburg, MD) respectively.

### Cell Proliferation Assay

Cell proliferation was measured by MTS assay (CellTiter 96 Aqueous One Solution Cell Proliferation assay) (Promega, Madison, WI). Briefly, 1×10^3^ cells were counted using a hemocytometer and were seeded in each well of 96-well microtiter plates. At the indicated time points, MTS solution was added to each well, followed by incubation for 20 minutes at 37°C in the dark, and the percentage of viable cells were quantified by measuring the absorbance at 490 nm using a microtiter culture plate reader. The absorbance at 490 nm for day 1 was set as one, and the relative proliferation rate for each day was calculated as values relative to day1.

### Prostaglandin Extraction and LC-MS/MS

All prostanoids and deuterated 13,14-dihydro-15-keto-PGE_2_ standard (added to homogenized samples as an internal control) were obtained from Cayman Chemical Co. Briefly, samples were first prepared in methanol (Fluka, Methanol LC-MS Chromasolv) (Sigma-Aldrich) and separated on C18 solid-phase extraction (SPE) cartridges (6 mL) (Cayman Chemical). The C18 cartridge was first preconditioned with 2 mL of methanol followed by 2 mL of water. Sample was then loaded onto the cartridge followed by a wash with 1 mL of water. Then, prostanoids were eluted with 1 mL of methanol. The eluted metabolites were then concentrated to a volume of 60 μL in MeOH for mass spectrometry analysis after vacuum-dried using ThermoSavant Integrated SpeedVac System SPD1010 (Thermo Scientific, Hemel Hempstead, UK). Mass spectrometry analyses were performed on LTQ-Orbitrap Velos (Thermo Scientific) mass spectrometer and chromatographic analyses were performed using Acquity UPLC systems (Waters, Hertsfordshire, UK).

### Cell Death Analysis

Cell death was detected using the Multi-Parameter Apoptosis Assay Kit (Cayman Chemical). Briefly, cells were counted and seeded in 96-well or 6-well plates. Cells without treatment or after treatment with the indicated drugs overnight were then washed with Cell-Based Assay Binding Buffer, and then incubated with Annexin V-FITC/7-Amino-Actinomycin D (7-AAD) double staining solution at room temperature in the dark for 10 minutes before resuspending in binding buffer for analysis. Fluorescence intensities were analyzed using SpectraMax M5 multi-detection microplate reader (Molecular Devices, Sunnyvale, CA) or on a FASCalibur flow cytometer (BD Bioscience, San Jose, CA) under FL1 (Annexin V-FITC) and FL3 (7-AAD) detectors. Annexin V-positive cells are defined as apoptotic, and Annexin V-7-AAD-double positive cells are defined as necrotic for flow cytometric analysis.

### Measurement of ROS Production

Cells without treatment or after treatment with the indicated drugs overnight were harvested in PBS and incubated in the presence of 10 μM 2’7’-dichlorodihydrofluorescein diacetate (H_2_DCFDA) (Invitrogen) in the dark at 37°C for 30 minutes. The shift in green fluorescence was measured in a FASCalibur flow cytometer (BD Bioscience) using the FL-1 detector and ROS production was determined from histogram data using CellQuest software (BD Bioscience). A total of 20,000 events were collected for each histogram.

### RNA Extraction and Quantitative Reverse Transcription-PCR (qRT-PCR)

RNA was isolated from cultured cells using TRI reagent (Ambion, Foster City, CA). The cDNA was reversely transcribed using random hexamers from 1 μg of total RNA using Transcription Reverse Transcriptase (Roche Applied Science) as per manufacturer’s instructions. The cDNA generated was used for qRT-PCR amplification using the LightCycler FastStart DNA Master ^PLUS^ SYBR Green I Kit (Roche Applied Science). The reaction was carried in the Light Cycler instrument (Roche Applied Science) and the PCR reaction was subjected to a melting curve analysis to verify the presence of a single amplicon using the Roche Molecular Biochemicals LightCycler Software (version 3.5). The mRNA levels were normalized to human cyclophilin expression level. Primer information is provided in (S1 Table) and the 2^-ΔΔCT^ relative quantification method was used to calculate the mean fold expression difference between the groups.

### Glutathione (GSH) assay

Cells were counted and seeded in 12-well plate. Cells without treatment or after treatment with the indicated drugs overnight were then collected and total GSH levels were measured using the ApoGSHTM GSH Colorimetric Detection Kit (BioVision) according to the manufacturer’s recommendations.

### Measurement of 13,14-dihydro-15-keto-PGE_2_ Production

Cells were counted and seeded in 24-well plates and culture medium was collected to measure the concentration of 13,14-dihydro-15-keto-PGE_2_ using Prostaglandin E Metabolite EIA Kit in accordance to the kit instructions (Cayman Chemical). The absorbance was measured using a microtiter culture plate reader at wavelength 405 nm. Logit B/B_0_ (standard bound/maximum bound) versus log PGEM concentration was plotted and the resulting linear regression fit was used for obtaining the concentration for each sample using the equation y = mx+b. The concentration for the control samples were set as one, and the relative levels of 13,14-dihydro-15-keto-PGE_2_ in PTGR2-silenced cells were calculated as values relative to the controls.

### Peroxisome Proliferator Response Element (PPRE) Luciferase Reporter Assay

Activation of PPRE was quantified by transiently co-transfecting cells with 300ng of Firefly luciferase reporter plasmid (PPRE×3-TK-Luc) and 100 ng of Renilla luciferase reporter plasmid (pRL-TK-Luc) [4] (internal standard) using Lipofectamine 2000 reagent (Life Technologies) according to the manufacturer’s instructions. After 24 hours of transfection, cells without treatment or after treatment with the indicated drugs for 3 hours were harvested for determination of luciferase activity. Luminescence was determined using the Dual-Glo Luciferase Assay Kit (Promega) according to manufacturer’s instructions. Luminescence values were normalized with regard to transfection efficiency by the use of a ratio of Firefly luciferase to Renilla luciferase luminescence.

### Cell Viability Crystal Violet Stain

Cells were counted and seeded in 24-well plates. After treatment with the indicated drugs overnight, cells were washed with PBS, fixed with 10% formalin for 30 minutes, and stained with 0.05% crystal violet for 30 minutes at room temperature. The relative levels of stain intensity to DMSO control were quantified by washing the stain with isopropanol and measuring the absorbance at 590 nm.

### Statistical Analysis

Statistical analyses were performed using the Student’s *t*-test with GraphPad Prism 4 (GraphPad Software, Inc., La Jolla, CA), and data are expressed as mean ± standard error (SE). The correlation between tumor PTGR2 stain and differentiation status and clinical stage parameters were analyzed using the Cochran-Mantel-Haenszel statistics implemented using the SAS procedure FREQ. *P<0*.*05* was considered a statistically significant difference.

## Results

### *PTGR2* is over-expressed in human pancreatic ductal adenocarcinoma

**Fig 1:**
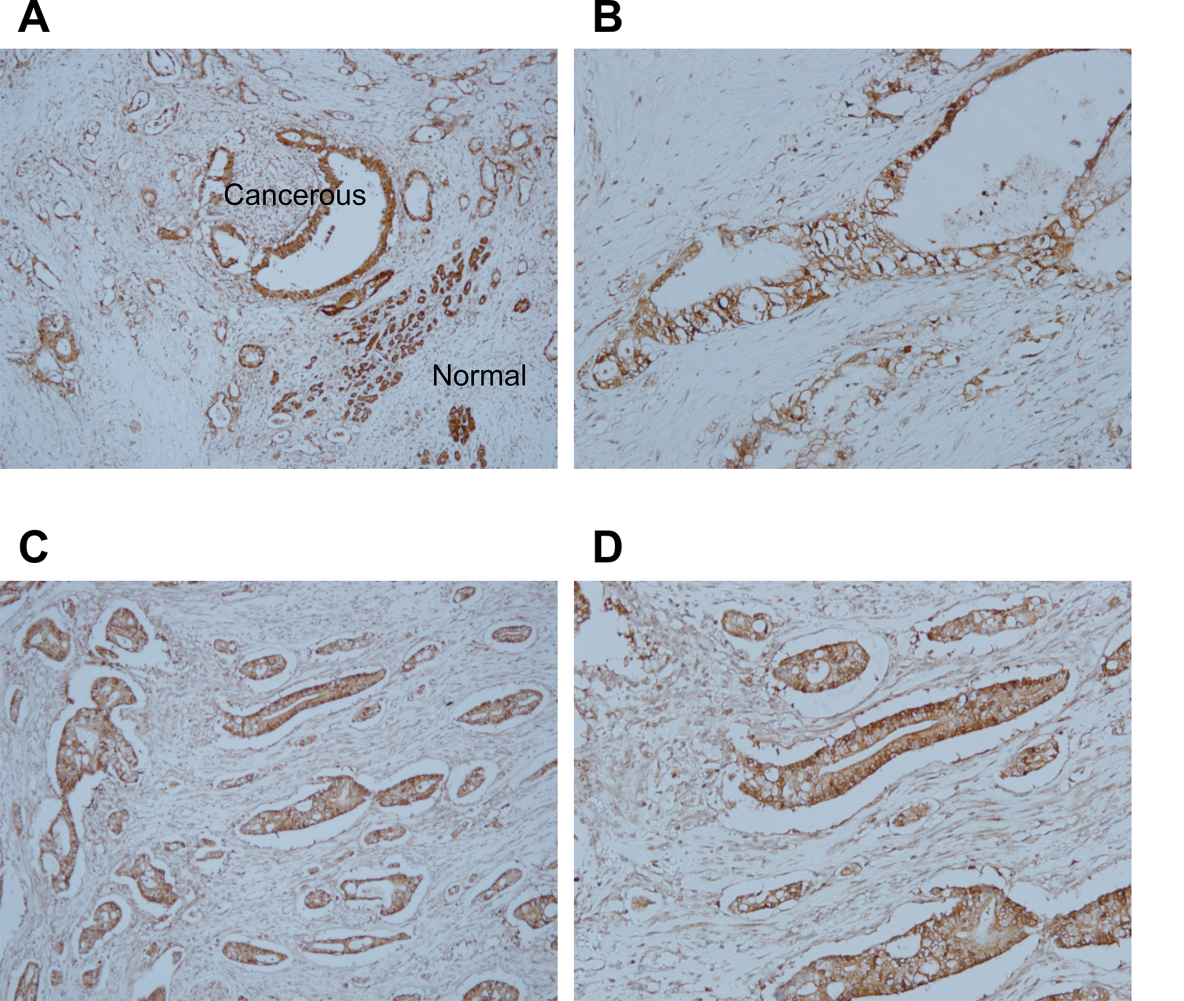
*PTGR2* protein expression in human pancreatic ductal adenocarcinoma tissues. **(A** and **C)** Two representative tissue sections (obtained from 76 patients) of pancreatic ductal adenocarcinoma tissues with adjacent normal tissues. Immunohistochemical staining was performed using a specific anti-PTGR2 antibody. Tumor regions were stained positive for PTGR2 (dark brown) whereas majority of the adjacent normal tissue cells were stained negative for PTGR2. Magnification X100. (**B** and **D)** Higher magnifications of **(A)** and **(C)** respectively for PTGR2-positive regions. Magnification 200X.)

Previously, we showed clinical significance for the high PTGR2 stain intensity in tumor areas relative to adjacent non-tumor areas in human gastric tumor tissues [18]. In the human protein atlas website (www.proteinatlas.org), we found PTGR2 expression in pancreatic cancer tissues but absent in normal tissues as well. To further dissect the role of PTGR2 in cancer biology, we examined the expression of PTGR2 in pancreatic ductal adenocarcinoma specimens from 76 patients using immunohistochemical staining. We found that majority (85.5%) of the pancreatic ductal adenocarcinoma tissues were stained positive for PTGR2 expression but not expressed in the adjacent normal parts (Fig 1), suggesting PTGR2 may play a general oncogenic role not restricted to previously reported gastric cancer. However, in patients with pancreatic ductal adenocarcinoma (PDCA), tumor-part PTGR2 stain intensity was not significantly associated with differentiation status or clinical stage (S2 Table).

### *PTGR2* affects growth of pancreatic cancer cells capable of self-producing prostaglandins

**Fig 2:**
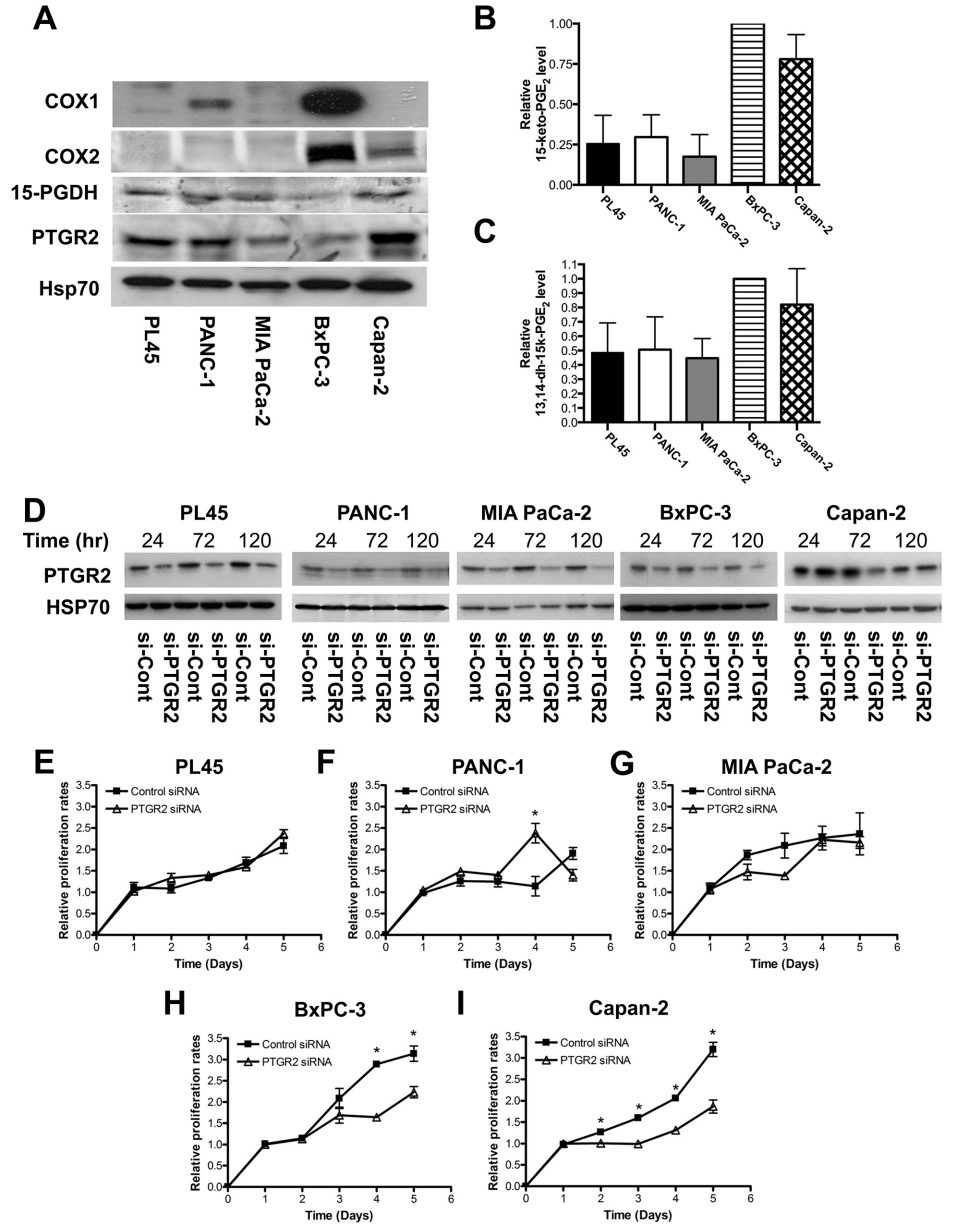
Silencing of *PTGR2* suppressed growth rates of pancreatic cancer cells capable of self-producing abundant prostaglandins. **(A)** Western blot analysis of expression levels of COX1, COX2, 15-PGDH, and PTGR2 in PL45, PANC-1, MIA PaCa-2, BxPC-3, and Capan-2 cells. Hsp70 served as a loading control. **(B** and **C**) Relative levels of intracellular **(B)** 15-keto-PGE_2_ and **(C)** 13,14-dihydro-15keto-PGE_2_ isolated from various pancreatic cancer cell lines. The concentrations of 15-keto-PGE_2_ and 13,14-dihydro-15keto-PGE_2_ extracted from BxPC-3 were set as 1, and the relative levels of 15-keto-PGE_2_ and 13,14-dihydro-15-keto-PGE_2_ from other cell lines were presented as values relative to BxPC-3 cells. Prostaglandins were isolated and analyzed by LC-MS/MS. The results are the average of 4 independent experiments. **(D)** Efficiency of siRNA-mediated *PTGR2* silencing. PTGR2 protein expression in PL45, PANC-1, MIA PaCa-2, BxPC-3 and Capan-2 cells was detected by Western blot analysis. Hsp70 served as a loading control. Time indicated hours post-siRNA transfection. **(E**–**I**) Relative proliferation rates of **(E)** PL45 **(F)** PANC-1 **(G)** MIA PaCa-2 **(H)** BxPC-3 and **(I)** Capan-2 si-PTGR2 cells as compared to si-Control cells. Cell proliferation was evaluated by MTS assay at the indicated time points. 48 hours post-transfection was set as Day 1. The values were obtained from 2 independent experiments each done in triplicate. Data are presented as the mean ± SE. * P < 0.05, Student’s t-test.

We next examined the expressions of major enzymes in the prostaglandin E_2_ pathway, including COX1, COX2, 15-PGDH and PTGR2, as well as the level of 15-keto-PGE_2_, in different pancreatic cancer cell lines. All five pancreatic cancer cell lines expressed 15-PGDH and PTGR2. However, only BxPC-3 and Capan-2 expressed COX2, and only PANC-1 and BxPC-3 expressed COX1 (Fig 2A). Furthermore, BxPC-3 and Capan-2, which expressed highest level of COX proteins, had noticeably higher levels of the PTGR2 substrate 15-keto-PGE_2_ and the PTGR2 product 13,14-dihydro-15-keto-PGE_2_ than in other cell lines including PL45, PANC-1 and MIA PaCa-2 (Fig 2B and 2C).

We then used siRNA approach to silence PTGR2 expression (Fig 2D). Interestingly, silencing of *PTGR2* significantly suppressed the growth rates of BxPC-3 and Capan-2 while the growth rates of PL45, PANC-1 and MIA PaCa-2 were unaffected (Fig 2E and 2F). Since BxPC-3 and Capan-2 could self-generate highest level of prostaglandins, we suspected that the biological outcomes resulted from silencing of *PTGR2* were related to the disturbance of prostaglandin metabolism.

### Silencing of *PTGR2* increased 15-keto-PGE_2_ levels and promoted pancreatic cancer cell death with increased ROS production

**Fig 3:**
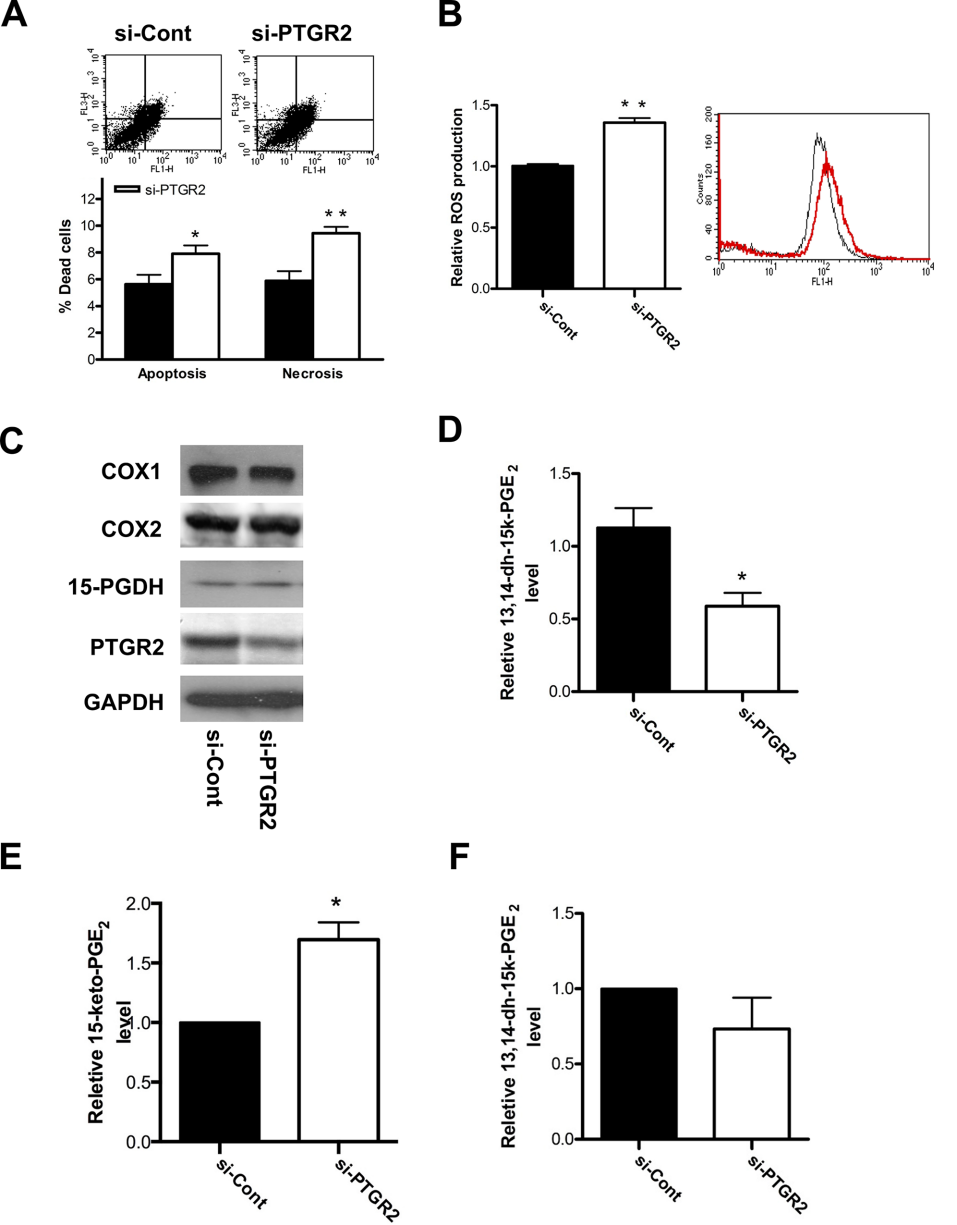
Silencing of *PTGR2* promoted cell death and ROS production and induced 15-keto-PGE_2_ in BxPC-3 cells. **(A)** The percentage of dead cells in si-PTGR2 BxPC-3 cells as compared to si-Control cells was evaluated by Annexin V and 7-AAD staining. The flow cytometry plots show annexin V-FITC binding (FL1-H) and 7-AAD staining (FL3-H). The bar graph distinguish dead cells as apoptotic or necrotic. The results are the average of 4 independent experiments each done in triplicate. (**B)** Relative ROS production in si-PTGR2 (red profile) BxPC-3 cells as compared to si-Control (black profile) cells. ROS was detected using H_2_DCF dye and flow cytometry. The results are the average of 3 independent experiments each done in triplicate. (**C)** Western blot analysis of the expression levels of COX1, COX2, 15-PGDH and PTGR2 in si-PTGR2 BxPC-3 cells. GAPDH served as a loading control. **(D)** Relative production of 13,14-dihydro-15-keto-PGE_2_ in si-PTGR2 BxPC-3 cells as compared to si-Control cells. The concentration for si-Control cells was set as 1, and the relative levels of 13,14-dihydro-15-keto-PGE_2_ in si-PTGR2 cells was presented as values relative to the control. The values were obtained from 2 independent experiments each done in triplicate. **(E** and **F)** Relative levels of intracellular **(E)** 15-keto-PGE_2_ and **(F)** 13,14-dihydro-15-keto-PGE_2_ isolated from si-PTGR2 BxPC-3 cells as compared to si-Control cells. The concentrations of 15-keto-PGE_2_ and 13,14-dihydro-15-keto-PGE_2_ extracted from si-Control cells were set as 1, and the relative levels of 15-keto-PGE_2_ and 13,14-dihydro-15-keto-PGE_2_ from si-PTGR2 cells were presented as values relative to the control. Prostaglandins were isolated and analyzed by LC-MS/MS. The results are the average of 3 independent experiments. Data are presented as the mean ± SE. * P < 0.05, ** P < 0.01, Student’s t-test.

Similar to those observed in gastric cancer cells [18], annexin V-7AAD binding assay and flow cytometric H_2_-DCFDA staining further showed that *PTGR2*-silenced BxPC-3 exerted elevated percentage of both apoptotic and necrotic cells (Fig 3A) as well as induced ROS production (Fig 3B). Similar outcomes were observed in Capan-2 cells, although to a lesser extent (S1A and S1B Fig).

We further found that silencing of *PTGR2* also did not affect the protein levels of COX1, COX2 and 15-PGDH in both BxPC-3 (Fig 3C) and Capan-2 cells (S2A Fig). Thus we next examined the levels of PTGR2 substrate and product after *PTGR2* silencing. Not only did *in vitro* immunoassay show reduced level of PTGR2 product 13,14-dihydro-15-keto-PGE_2_ (Fig 3D and S2B Fig), LC/MS quantification further confirmed the higher level of 15-keto-PGE_2_ and reduced level of 13,14-dihydro-15-keto-PGE_2_ in *PTGR2*-silenced pancreatic cancer cells as compared to controls (Fig 3E and 3F and S2C and S2D Fig).

### Suppressed cell viability after silencing of *PTGR2* is mediated through 15-keto-PGE_2_ and is not entirely dependent on PPARγ activity

**Fig 4:**
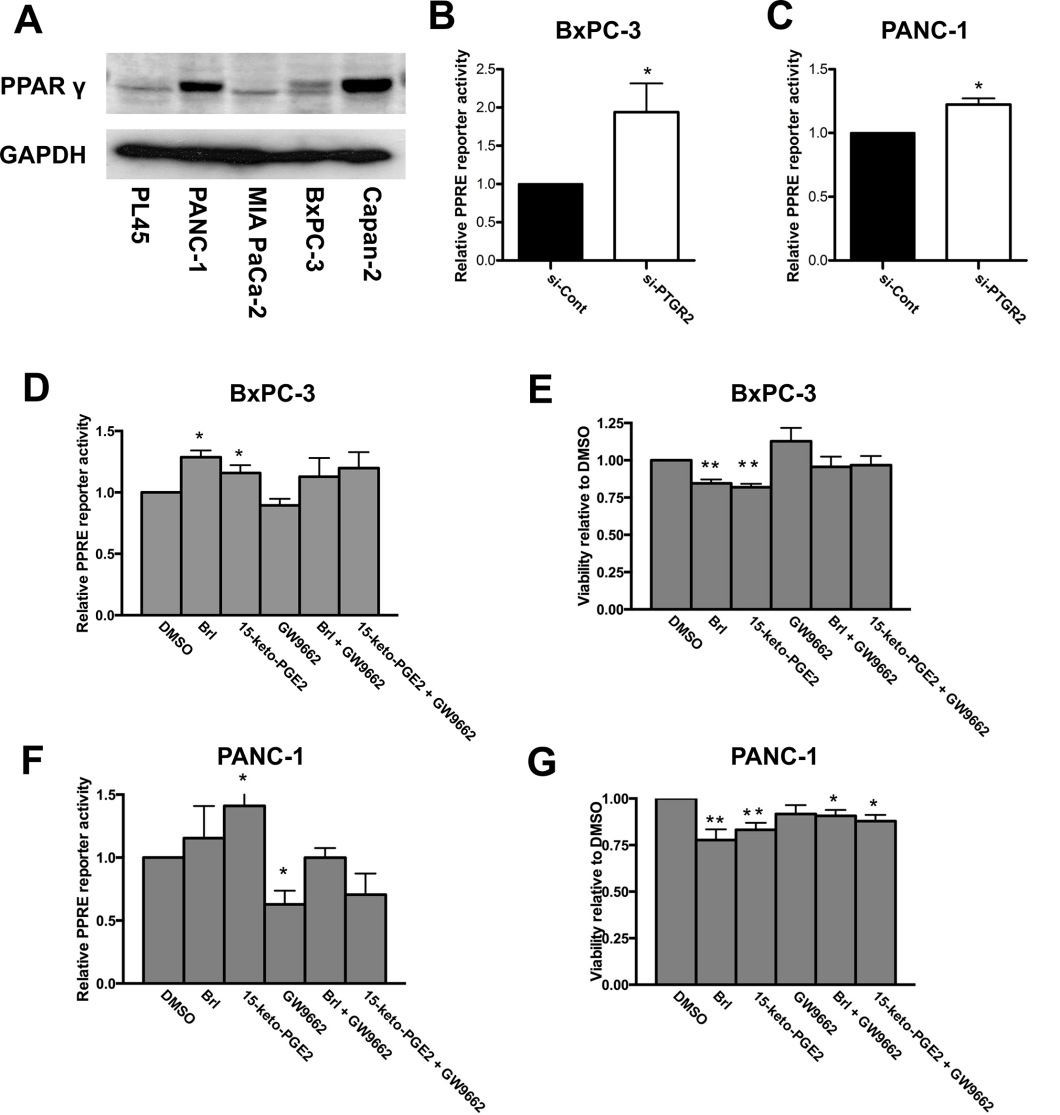
Suppressed cell viability induced by 15-keto-PGE_2_ is not entirely dependent on PPARγ activity. **(A)** Western blot analysis of the expression level of PPARγ in PL45, PANC-1, MIA PaCa-2, BxPC-3, and Capan-2 cells. GAPDH served as a loading control. **(B**, **C, D** and **F)** After **(B** and **C)** siRNA treatment or **(D** and **F)** drug treatment for 3 hours, cells were further transfected with reporter plasmids and luciferase activity was measured in **(B** and **D)** BxPC-3 and **(C** and **F)** PANC-1 cells. Luciferase activity for si-Control or DMSO-treated cells was set as 1, and the relative luciferase activity for si-PTGR2 or drug-treated cells was presented as value relative to the control. The results for **(B** and **C)** are the average of 4 independent experiments each done in triplicate and the results for **(D** and **F)** are the average of 3 independent experiments each done in duplicates. **(E** and **G)** Cell viability of **(E)** BxPC-3 and **(G)** PANC-1 cells was performed by crystal violet stain and quantified by measuring the absorbance at 590nm after isopropanol wash. Cells were treated with DMSO or 15-keto-PGE_2_ overnight. The viability of cells treated with DMSO was set as 1 and the relative level of viability of drug-treated cells was presented as value relative to the control. The results are the average of 4 independent experiments. Data are presented as the mean ± SE. * P < 0.05, ** P < 0.01, Student’s t-test.

Since past studies have reported that 15-keto-PGE_2_ could induce cell death and is a natural PPARγ ligand and that activation of PPARγ inhibited cancer cell proliferation through increasing ROS [4, 44, 45], we suspected that the tumor-suppressor effect of *PTGR2* silencing was mediated through increased 15-keto-PGE_2_ and possibly through PPARγ. We next examined the expression of PPARγ in all five pancreatic cell lines. Two cell lines (PANC-1 and Capan-2) exhibited higher expression level while the other three cell lines (PL45, MIA PaCa-2, and BxPC-3) had lower expression level (Fig 4A). Silencing *PTGR2* induced PPARγ transcriptional activity in cell lines expressing either low (BxPC-3) or high (PANC-1) PPARγ (Fig 4B and 4C).

To elucidate if the biological outcomes observed from silencing of *PTGR2* were related to PPARγ activity, we first treated BxPC-3 with synthetic PPARγ agonist Brl and 15-keto-PGE_2_ alone or together with the irreversible PPARγ antagonist GW9662 and performed PPRE reporter assay to ensure that the drugs we used worked properly to induce or to block PPARγ transcriptional activity. PPRE reporter assay showed that both Brl, 15-keto-PGE_2_ and GW9662 worked properly, although they seemed to work better in PANC-1 cells (Fig 4D and 4F), possibly due to the fact that BxPC-3 already self-produce abundant 15-keto-PGE_2_ and thus the drugs were not as effective. Consistently, treatment with Brl or 15-keto-PGE_2_ also suppressed cell viability in both BxPC-3 and PANC-1 cells. GW9662 seemed to be able to reverse the suppressive effect of 15-keto-PGE_2_ and Brl in BxPC-3 slightly, but was ineffective in PANC-1 cells (Fig 4E and 4G). Our data suggested that the cell death-promoting effect of 15-keto-PGE_2_ is mediated partly but not entirely through PPARγ activation.

### Induced cell death and ROS production in *PTGR2*-silenced cells are due to a defective antioxidative defense system

The biological functions of PTGR2 we observed in pancreatic cancer cells seemed to be similar to gastric cancer cells. However, it is still unclear how PTGR2 affects cellular ROS production, and in turn induced cell death [18]. Thus, we next attempted to investigate why manipulating PTGR2 expression, which altered the concentration of its substrate 15-keto-PGE_2_, affected ROS and cell death.

**Fig 5:**
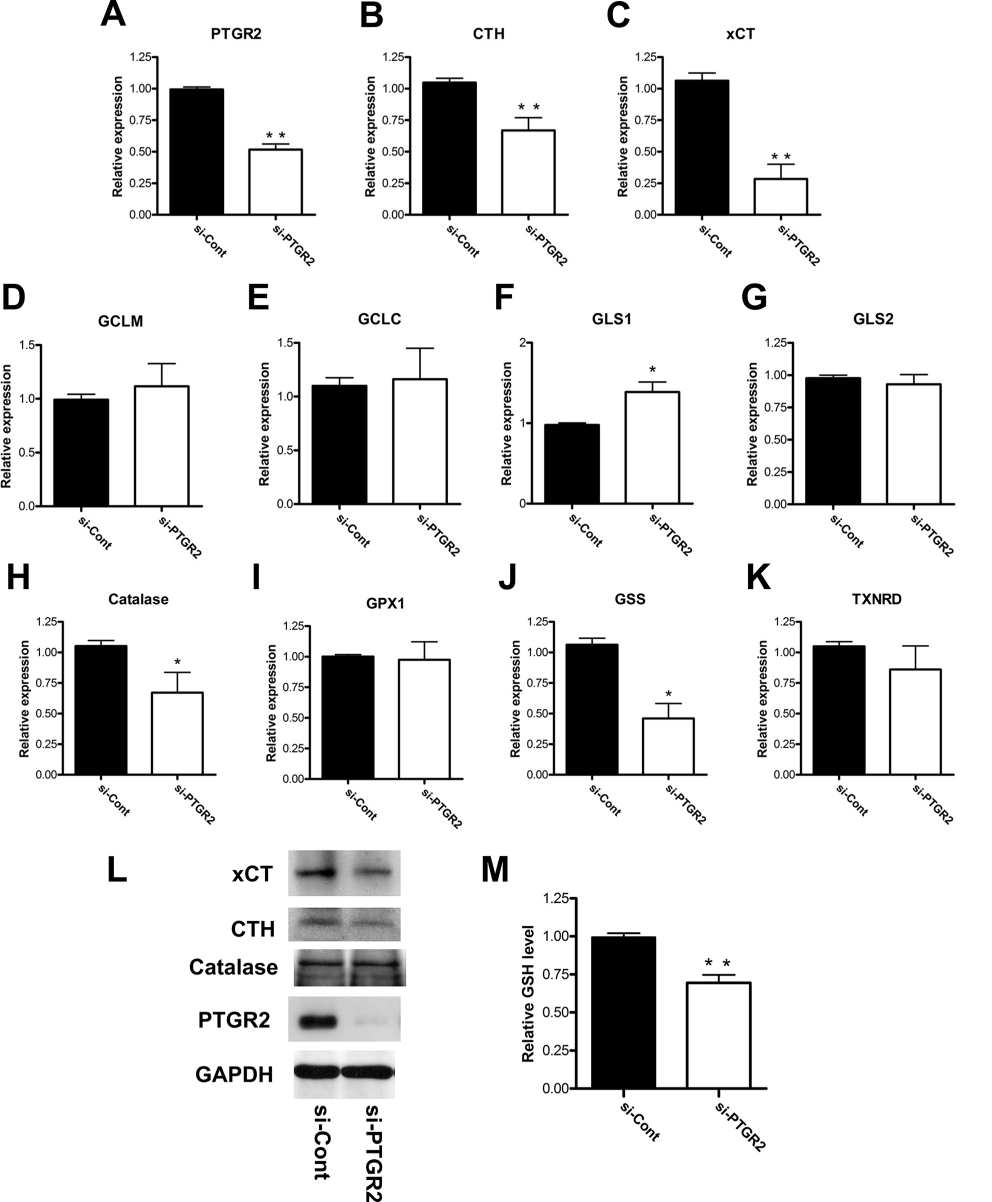
Silencing of *PTGR2* suppressed expression levels of antioxidative genes xCT and CTH and total cellular glutathione level in BxPC-3 cells. **(A**–**K)** Relative mRNA expression levels of (**A**) PTGR2 (**B**) CTH (**C**) xCT (**D**) GCLM (**E**) GCLC (**F**) GLS1 (**G**) GLS2 (**H**) Catalase (**I**) GPX1 (**J**) GSS and (**K**) TXNRD in si-PTGR2 BxPC-3 cells as compared to si-Control cells. Total RNA was harvested and subjected to qRT-PCR analysis and the mRNA levels were normalized to human cyclophilin expression level. mRNA expression levels in si-Control cells were set as 1 and the relative mRNA expression levels in si-PTGR2 cells were presented as values relative to the control. The results are the average of 3 independent experiments each done in triplicate. **(L)** Western blot analysis of the expression levels of xCT, CTH, Catalase and PTGR2 in si-PTGR2 BxPC-3 cells. GAPDH served as a loading control. **(M)** Total GSH level in si-PTGR2 BxPC-3 cells as compared to si-Control cells. The GSH level in si-Control cells was set as 1 and the GSH level in si-PTGR2 cells was presented as values relative to the control. The results are the average of 3 independent experiments each done in triplicate. Data are presented as the mean ± SE. * P < 0.05, ** P < 0.01, Student’s t-test.

It is well known that cancer cells have higher level of ROS to contribute to their faster growth rates and tumor formation, and thus they also exert higher antioxidants to regulate ROS to levels advantages to them but not detrimental [19, 46]. The three major antioxidant pathways include glutathione-dependent pathway, thioredoxin-dependent pathway, and catalase [19]. Thus, we first examined if silencing of *PTGR2* altered the expression levels of major genes regulating cellular oxidative stress. qRT-PCR analyses of a total of 10 genes showed that only the expressions of cystathionine gamma-lyase (CTH), solute carrier family 7 member 11 (xCT) and catalase were reduced in both *PTGR2*-silenced BxPC-3 (Fig 5A–5K) and Capan-2 cells (S3A–S3F Fig) as compared to the control cells. We further confirmed the protein expressions of these three genes and found that only xCT and CTH expressions were suppressed after silencing of PTGR2. Catalase on the other hand did not show a differed protein expression level (Fig 5L and S3G Fig). Since xCT and CTH are important in providing cellular cysteine supply for synthesizing glutathione [32, 34, 47], we further examined and observed a significant decreased glutathione concentration in *PTGR2*-silenced BxPC-3 cells as compared to the control cells (Fig 5M). Thus, the elevated ROS in *PTGR2*-silenced cells are possibly due to a defective antioxidative defense system.

### *PTGR2* silencing induces ROS-dependent cell death by reducing cellular glutathione level through manipulating *xCT* and *CTH* as a result of excess 15-keto-PGE_2_

**Fig 6:**
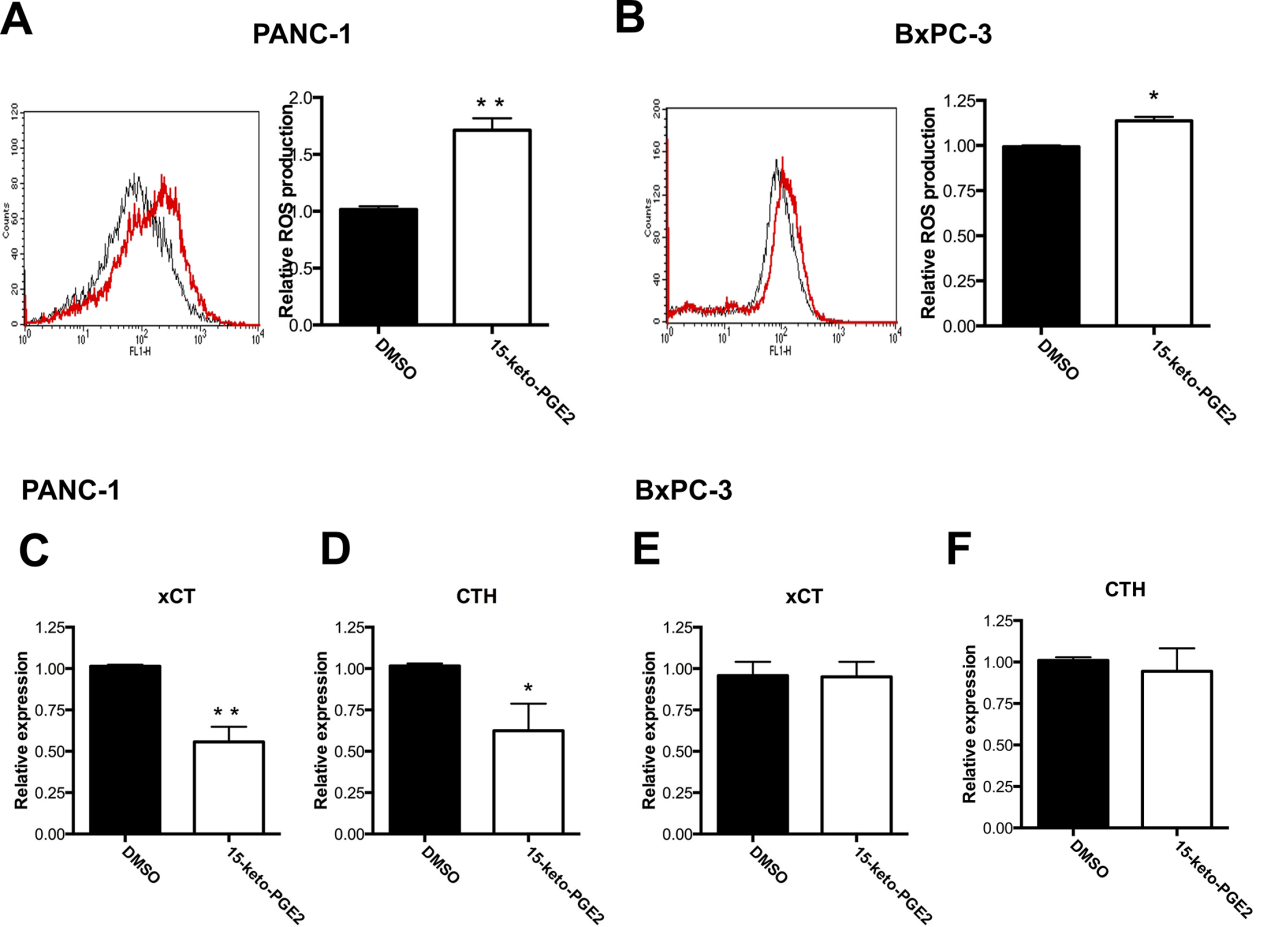
Effects of 15-keto-PGE_2_ on ROS production and antioxidative genes *xCT* and *CTH*. **(A** and **B)** Relative ROS production in 15-keto-PGE_2_-treated (red profile) (**A**) PANC-1 and (**B**) BxPC-3 cells as compared to DMSO-treated (black profile) cells. Cells were treated with DMSO or 15-keto-PGE_2_ overnight and ROS was detected using H_2_DCF dye and flow cytometry. The results are the average of 3 independent experiments each done in triplicate. **(C–F)** Relative mRNA expression levels of (**C** and **E**) xCT and (**D** and **F**) CTH in 15-keto-PGE_2_-treated (**C** and **D**) PANC-1 or (**E** and **F**) BxPC-3 cells as compared to DMSO-treated cells. Cells were treated with DMSO or 15-keto-PGE_2_ overnight and total RNA was harvested and subjected to qRT-PCR analysis. The mRNA levels were normalized to human cyclophilin expression level. mRNA expression levels in DMSO-treated cells were set as 1 and the relative mRNA expression levels in 15-keto-PGE_2_-treated cells were presented as values relative to the control. The results are the average of 3 independent experiments each done in triplicate. Data are presented as the mean ± SE. * P < 0.05, ** P < 0.01, Student’s t-test.

We next treated cells with 15-keto-PGE_2_ to see if similar outcomes were observed as in *PTGR2*-silenced cells. Treatment of PANC-1 cells with 15-keto-PGE_2_ also resulted in significantly elevated ROS production (Fig 6A). Similar finding was obtained in BxPC-3 although the difference was not as statistical significant (Fig 6B). qRT-PCR further showed that although treatment with 15-keto-PGE_2_ did not affect *xCT* and *CTH* expression levels in BxPC-3 cells (Fig 6E and 6F), we observed significantly reduced *xCT* and *CTH* expressions in PANC-1 cells (Fig 6C and 6D).

**Fig 7:**
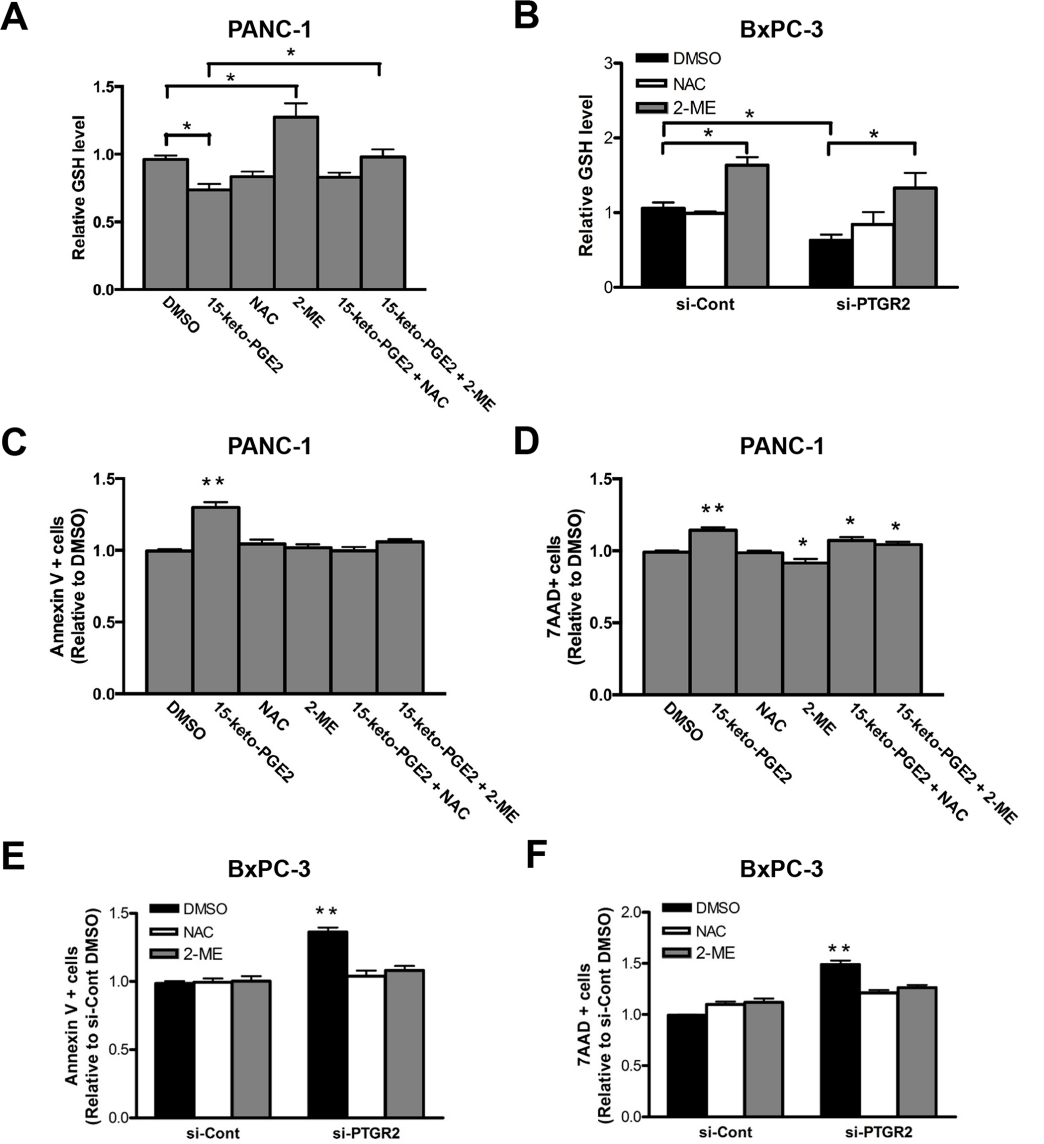
Induced cell death by 15-keto-PGE_2_ in PANC-1 cells or in *PTGR2*-silenced BxPC-3 cells could be reversed by restoring GSH level or intracellular cysteine supply. **(A)** Total GSH level was measured in PANC-1 cells treated with the indicated drugs overnight. The GSH level in DMSO-treated cells was set as 1 and the GSH level in other drug-treated cells was presented as values relative to the control. The results are the average of 5 independent experiments each done in duplicate. **(B**) After si-RNA treatment in BxPC-3 cells, cells were further treated with DMSO, NAC or 2-ME overnight and total GSH level was measured. The GSH level in si-Control cells treated with DMSO was set as 1 and the GSH level in NAC, 2-ME and si-PTGR2 cells was presented as values relative to the control. The results are the average of 3 independent experiments each done in duplicate. **(C** and **D)** Levels of (**C**) apoptotic and (**D**) necrotic cells in PANC-1 cells treated with the indicated drugs overnight were evaluated by Annexin V and 7-AAD staining followed by flow cytometry. The level of dead cells treated with DMSO was set as 1 and the level of dead cells in other drug-treated cells was presented as values relative to the control. The results are the average of 4 independent experiments each done in triplicate. **(E** and **F)** After si-RNA treatment, BxPC-3 cells were further treated with DMSO, NAC or 2-ME overnight and the level of (**E**) apoptotic and (**F**) necrotic cells was evaluated by Annexin V and 7-AAD staining followed by flow cytometry. The level of dead cells in si-Control cells treated with DMSO was set as 1 and the level of dead cells in NAC, 2-ME and si-PTGR2 cells was presented as values relative to the control. The results are the average of 4 independent experiments each done in triplicate. Data are presented as the mean ± SE. * P < 0.05, ** P < 0.01, Student’s t-test.

Next, we thought to see if 15-keto-PGE_2_ could also induce cell death as did silencing *PTGR2*, and if so, whether the cell death induced is caused by excess ROS accumulated as a result of reduced GSH due to down-regulated *xCT* and *CTH*. NAC is a well-known ROS scavenger and can augment intracellular GSH concentration. Another compound 2-ME is commonly used to restore intracellular cysteine supply for GSH synthesis when xCT is blocked [34, 37, 48]. Thus, we treated PANC-1 cells with 15-keto-PGE_2_, NAC, and 2-ME alone or in combinations and assayed for GSH concentration and cell death. Treatment with 15-keto-PGE_2_ caused a significantly reduced GSH level. This effect of 15-keto-PGE_2_ was reversed when co-treatment with NAC or 2-ME. 2-ME alone also effectively induced GSH concentration (Fig 7A). For the effect on cell death, although the percentages of necrotic cells were not affected significantly, co-treatment with NAC and 2-ME also reversed the elevated level of apoptotic cells induced by 15-keto-PGE_2_ (Fig 7C and 7D).

Our study showed that BxPC-3 did not respond as well to 15-keto-PGE_2_ as did PANC-1, possibly due to its high levels of self-produced prostaglandins. Thus, we confirmed the effect of NAC and 2-ME directly using *PTGR2*-silenced BxPC-3 cells. In control or *PTGR2*-silenced cells, treatment with NAC and especially 2-ME induced GSH level. Importantly, when comparing to cells treated with control siRNA, the reduced GSH level after silencing of *PTGR2* was reversed when treating cells with NAC or 2-ME (Fig 7B). Moreover, induced apoptotic and necrotic cells after silencing of *PTGR2* was rescued after treatment with NAC and 2-ME (Fig 7E and 7F). Taken together, our data suggested that silencing *PTGR2*, which resulted in excess 15-keto-PGE_2,_ induced ROS-dependent cell death by affecting cellular glutathione level through manipulating *xCT* and *CTH* expressions.

## Discussion

In the present study, we demonstrated that the oncogenic potency of PTGR2 is not restricted to gastric cancer but is also observed in pancreatic cancer. PTGR2 is strongly stained in human pancreatic ductal adenocarcinoma tissue. Silencing of *PTGR2* suppressed pancreatic cancer cell growth and induced cancer cell death through increased 15-keto-PGE_2_ and ROS levels. These effects are possibly mediated through suppressed *xCT* and *CTH* expressions, causing depleted GSH level and disrupting antioxidative defense. To our knowledge, this is the first study demonstrating PTGR2/15-keto-PGE_2_ plays a role in antioxidant signaling.

In our study, silencing of *PTGR2* only affected the proliferation rate of BxPC-3 and Capan-2, both of which can self-generate abundant prostaglandins due to the high expression levels of COX proteins. It is now well established that the cells within the stromal compartment surrounding pancreatic cancer cells contribute to the progression of tumor because some pancreatic cancer cells require tumor microenvironment to provide prostaglandin metabolites and nutrients [1, 49–51]. This heterogeneity of pancreatic cancer phenotypes also reflected in our study as silencing *PTGR2*, which in turn disturbed prostaglandin metabolism, did not show noticeable effect on cell lines that cannot self-generate prostaglandins, including PL45, PANC-1 and MIA PaCa-2. Although PANC-1 expressed COX1, past study showed that COX2 inhibitor but not COX1 inhibitor could effectively suppress growth of pancreatic cancer [3]. Since there are abundant cancer-associated fibroblasts (CAFs) in the tumor stromal area under physiological condition, we speculate that under co-culture condition with CAFs, suppressed growth rate or induced cell death we observed in *PTGR2*-silenced pancreatic cancer cells (BxPC-3 and Capan-2) could be lost because the decrease in 15-keto-PGE_2_ after silencing of *PTGR2* could be provided by the tumor microenvironment. As the biological outcomes obtained from silencing of *PTGR2* were related to the disturbance of prostaglandin metabolism, this also suggested the importance of PTGR2 enzymatic activity, which greatly affects the concentrations of its substrate and product.

Numerous studies have already extensively documented the role of prostaglandins in cancer biology [7, 8, 52–54]. Besides our previous finding that PTGR2, an enzyme degrading 15-keto-PGE_2_, modulates cell death and tumor transformation of gastric cancer cells [18], recent studies also showed an emerging role of 15-PGDH, an enzyme generating 15-keto-PGE_2_, as a tumor suppressor in different cancers [55–60]. Originally, little is known about the tumor suppressive role of 15-keto-PGE_2_ except the proposition by Lalier et al. that 15-keto-PGE_2_ could induce cell death by activating the proapoptotic protein Bax [44]. Recently, two studies by Lu et al. further demonstrated that the growth inhibition and tumor suppressive role of 15-PGDH on hepatocellular carcinoma and intrahepatic cholangiocarcinoma cells were in fact mediated through its enzymatic product 15-keto-PGE_2_, which in turn activated PPARγ/p21^WAF1/Cip1^ and PPARγ/Smad2/3/TAP63 signaling cascades respectively [61, 62]. In both studies, the 15-PGDH/15-keto-PGE_2_-mediated PPARγ transcription activities were inhibited by overexpression of *PTGR2*. These studies substantiate our findings on the functional significance of PTGR2/15-keto-PGE_2_ in cancer biology.

Because 15-keto-PGE_2_ is an endogenous PPARγ ligand, we also attempted to investigate whether the biological outcomes observed from silencing of *PTGR2* were related to PPARγ activity. In our study, silencing *PTGR2* or addition of 15-keto-PGE_2_ both induced PPARγ transcriptional activity. Thus, PPARγ downstream signaling must play certain roles in cancer progression in our study models. However, addition of the selective and irreversible PPARγ antagonist GW9662 did not completely reverse the 15-keto-PGE_2_-induced cell death. Furthermore, PPARγ synthetic ligand Brl also did not affect gene expression levels of *xCT* and *CTH* as did 15-keto-PGE_2_ (data not shown). These suggest that the role of 15-keto-PGE_2_ specifically in the regulation of antioxidative signaling through xCT and CTH may not require PPARγ in pancreatic cancer cells.

Although recently PPARγ has been shown to inhibit cancer cell proliferation by reducing GSH level leading to excess ROS-induced cell-cycle arrest [45] and extensive studies have shown important roles of PPARγ in pancreatic cancer [41, 63–66], numerous studies have also documented tumor suppressive roles of PPARγ ligands independent of PPARγ activation, including but not restricted to an induction of apoptosis and ROS [67, 68]. Furthermore, recent study by Min et al. even documented the existence of both PPARγ-dependent and PPARγ-independent function of the PPARγ ligand MCC-555 in pancreatic cancer, where the induction effect of the cell cycle inhibitor p21 and the pro-apoptotic protein NAG-1 by MCC-555 is independent of PPARγ signaling, but the suppression of cyclin D1 by MCC-555 is dependent on PPARγ signaling [69]. Interestingly, Kristiansen et al. suggested PPARγ as a prognostic marker for pancreatic cancer as they found strong PPARγ expression in pancreatic ductal adenocarcinoma with shorter patients’ survival times. Nonetheless, there were no correlations with PPARγ downstream target gene expressions; many target genes were even under-expressed when PPARγ expression was highly induced. This discrepancy along with PPARγ-independent tumor suppressive roles of PPARγ ligands prompted the possibility of an impaired function of PPARγ in pancreatic cancer [43]. It is without doubt that PPARγ is an important player in cancer biology, and although our data suggested a PPARγ-independent inhibition of *xCT* and *CTH* that lead to induced ROS and apoptosis by 15-keto-PGE_2,_ we cannot completely rule out the possibility of PPARγ-dependent function by 15-keto-PGE_2_ in our study.

Manipulating *PTGR2* expression affects both the level of its substrate 15-keto-PGE_2_, as well as the level of its product 13,14-dihydro-15-keto-PGE_2_. Since we did not completely silence *PTGR2* in our experiments and the exogenous 15-keto-PGE_2_ we added would be metabolized into 13,14-dihydro-15-keto-PGE_2_, it is also possible that the biological outcomes we observed in this study came from 13,14-dihydro-15-keto-PGE_2_. Nonetheless, treatment of pancreatic cancer cells with 13,14-dihydro-15-keto-PGE_2_ induced cell growth slightly but was ineffective in affecting cell death or ROS level (data not shown). Although the ineffectiveness of 13,14-dihydro-15-keto-PGE_2_ could be due to its unstable nature as well as experimental factors such as small molecule concentrations or delivering approach, whether 13,14-dihydro-15-keto-PGE_2_ possess any biological function still needs to be clarified.

Besides PPARγ, plausible potential pathways lie in other transcriptional factor signaling. Both CTH and xCT are regulated by the stress-inducible transcriptional factor nuclear factor erythroid 2-related factor 2 (Nrf2), which is also one of the most important regulators of genes in the antioxidative signaling pathway, including GSH level [19, 70]. Furthermore, *xCT* gene expression can also be regulated by the transcriptional factor activating transcription factor 4 (ATF4) during oxidative stress while *CTH* expression is also critically regulated by the transcriptional factor Sp1 and by the PI3K/AKT pathway [71, 72]. A recent study also proposed that PI3K signaling regulates the expression of ATF4 as well [73]. As Nrf2 is overexpressed in pancreatic cancer and little is known about xCT and especially CTH regulation in tumor, further study is required to clarify how PTGR2/15-keto-PGE_2_ specifically regulates *CTH* and *xCT* expression, whether directly through oxidative stress-related transcriptional factors or through their upstream regulators.

In summary, we revealed the importance of PTGR2/15-keto-PGE_2_ in antioxidative signaling and in tumor cell death involving xCT and CTH in some of the pancreatic cancer cells. Although much work is still needed to clarify the underlying specific mechanisms, clinically we also found that majority of pancreatic ductal adenocarcinoma tissues were stained positive for PTGR2 expression, similar to what we observed in human gastric cancer. Recently, studies have suggested modulating the redox status of cancer cells as therapeutic approach [74–76]. Together with our clinical finding, the significance of PTGR2 in cancer biology as well as plausible target to improve therapeutic efficacy of existing cancer drugs is worth continuing and exploring.

## Supporting Information

S1 Table Primers used for quantitative real-time PCR. (DOCX)

S2 Table Correlation of *PTGR2* stain intensity with differentiation status and clinical stage in patients with pancreatic ductal adenocarcinoma (PDCA). (DOCX)

S1 Fig Silencing of *PTGR2* induced cell death and ROS production in Capan-2 cells. (DOCX)

S2 Fig Relative levels of 15-keto-PGE_2_ and 13,14-dihydro-15-keto-PGE_2_ in si-Control and si-PTGR2 Capan-2 cells. (DOCX)

S3 Fig Silencing of *PTGR2* suppressed expression levels of antioxidative genes *xCT* and *CTH* in Capan-2 cells. (DOCX)
