# Supplementary material for: Inhibition of Prostaglandin Reductase 2, a Putative Oncogene Overexpressed in Human Pancreatic Adenocarcinoma, Induces Oxidative Stress-Mediated Cell Death Involving xCT and CTH Gene Expressions through 15-Keto-PGE2
Source: PLoS One. 2016 Jan 28;11(1):e0147390. doi: 10.1371/journal.pone.0147390 (PMC4731085; doi:10.1371/journal.pone.0147390)
Supplement: S1 Table — (DOCX) [file pone.0147390.s001.docx]

**S1 Table. Primers used for quantitative real-time PCR**

| **Genes** | **Sense primers** | **Antisense primers** |
| --- | --- | --- |
| PTGR2  CTH  xCT  GCLM  GCLC  GLS1  GLS2  Catalase  GPX1  GSS  TXNRD  Cyclophilin | 5’-ttggcagctatctcaagtcg-3’  5’-agttcctggaatctaatccttgg-3’  5’-caaatgcagtggcagtgacc-3’  5’-cagtgggcacaggtaaaacc-3’  5’-ccctcctccaaactcagaca-3’  5’-cactgccctcccattacctag-3’  5’-acaccctcagcctcatgcat-3’  5’-actttgaggtcacacatgacatt-3’  5’-ctcttcgagaagtgcgaggt-3’  5’-ctctacggctcacccaatgc-3’  5’-ttgccactggtgaaagacca-3’  5’-gcatacgggtcctggcatcttgtcc-3’ | 5’-tgtgtttgctttcttctataattccaatac-3’  5’-atgctgtggatgagaggg-3’  5’-agacagcaaacacaccaccg-3’  5’-aatgcagtcaaatctggtggc-3’  5’-gcagtaccacaaacaccacata-3’  5’- gaagctcaagcatgggaacag -3’  5’-atggctcctgatacagctgactt-3’  5’-ctgaacccgattctccagca-3’  5’-gatgtcaggctcgatgtcaa-3’  5’-tcgtcggatcacatggatgtt-3’  5’-aggatgctccaacaaccagg-3’  5’-atggtgatcttcttgctggtcttgc-3’ |
