# Supplementary material for: Inhibition of Prostaglandin Reductase 2, a Putative Oncogene Overexpressed in Human Pancreatic Adenocarcinoma, Induces Oxidative Stress-Mediated Cell Death Involving xCT and CTH Gene Expressions through 15-Keto-PGE2
Source: PLoS One. 2016 Jan 28;11(1):e0147390. doi: 10.1371/journal.pone.0147390 (PMC4731085; doi:10.1371/journal.pone.0147390)
Supplement: S2 Table — (DOCX) [file pone.0147390.s002.docx]

S2 Table. Correlation of PTGR2 stain intensity with differentiation status and clinical stage in patients with pancreatic ductal adenomcarcinoma (PDCA)

| PTGR2 stain intensity | 0  (n=11) | 1  (n=19) | 2  (n=17) | 3  (n=29) | *P*-value |
| --- | --- | --- | --- | --- | --- |
| Differentiation status (well: moderate: poor) | 0:10:1 | 1:15:2 | 2:12:3 | 2:25:2 | 0.51 |
| Tumor size (grade 2:3:4) | 1:8:2 | 2:15:2 | 1:17:2 | 0:26:4 | 0.45 |
| Lymph node involvement (no: yes) | 1:10 | 8:11 | 9:10 | 10:20 | 0.44 |
| Distant metastasis (no: yes) | 10:1 | 19:0 | 19:0 | 28:0 | 0.14 |
| AJCC stage (1:2:3:4) | 0:8:2:1 | 1:16:2:0 | 0:17:2:0 | 0:8:2:2:0 | 0.72 |

AJCC: American Joint Committee on Cancer
