# Supplementary material for: Inhibition of Prostaglandin Reductase 2, a Putative Oncogene Overexpressed in Human Pancreatic Adenocarcinoma, Induces Oxidative Stress-Mediated Cell Death Involving xCT and CTH Gene Expressions through 15-Keto-PGE2
Source: PLoS One. 2016 Jan 28;11(1):e0147390. doi: 10.1371/journal.pone.0147390 (PMC4731085; doi:10.1371/journal.pone.0147390)
Supplement: S1 Fig — (DOCX) [file pone.0147390.s003.docx]

**S1 Fig. Silencing of *PTGR2* induced cell death and ROS production in Capa-2 cells.**

**(A)** The percentage of dead cells in si-PTGR2 Capan-2 cells as compared to si-Control cells was evaluated by Annexin V and 7-AAD staining. The flow cytometry plots show annexin V-FITC binding (FL1-H) and 7-AAD staining (FL3-H). The bar graph distinguish dead cells as apoptotic or necrotic. The results are the average of 3 independent experiments each done in triplicate. (**B)** Relative ROS production in si-PTGR2 (red profile) Capan-2 cells as compared to si-control (black profile) cells. ROS was detected using H_2_DCF dye and flow cytometry. The results are the average of 3 independent experiments each done in triplicate. Data are presented as the mean ± SE. * *P* < 0.05, ** *P* < 0.01, Student’s *t*-test.
