# Supplementary material for: Inhibition of Prostaglandin Reductase 2, a Putative Oncogene Overexpressed in Human Pancreatic Adenocarcinoma, Induces Oxidative Stress-Mediated Cell Death Involving xCT and CTH Gene Expressions through 15-Keto-PGE2
Source: PLoS One. 2016 Jan 28;11(1):e0147390. doi: 10.1371/journal.pone.0147390 (PMC4731085; doi:10.1371/journal.pone.0147390)
Supplement: S2 Fig — (DOCX) [file pone.0147390.s004.docx]

**S2 Fig. Relative levels of 15-keto-PGE_2_ and 13,14-dihydro-15-keto-PGE_2_ in si-Control and si-PTGR2 Capan-2 cells.**

**(A)** Western blot analysis of the expression levels of COX2, 15-PGDH and PTGR2 in si-PTGR2 Capan-2 cells. GAPDH served as a loading control. (**B)** Relative production of 13,14-dihydro-15-keto-PGE_2_ in si-PTGR2 Capan-2 cells as compared to si-Control cells. The concentration for si-Control cells was set as 1, and the relative levels of 13,14-dihydro-15-keto-PGE_2_ in si-PTGR2 cells was presented as values relative to the control. The values were obtained from 2 independent experiments each done in triplicate. (**C** and **D)** Relative levels of intracellular **(C)** 15-keto-PGE_2_ and **(D)** 13,14-dihydro-15-keto-PGE_2_ isolated from si-PTGR2 Capan-2 cells as compared to si-Control cells. The concentrations of 15-keto-PGE_2_ and 13,14-dihydro-15-keto-PGE_2_ extracted from si-Control cells were set as 1, and the relative levels of 15-keto-PGE_2_ and 13,14-dihydro-15-keto-PGE_2_ from si-PTGR2 cells were presented as values relative to the control. Prostaglandins were isolated and analyzed by LC-MS/MS. The results are the average of 3 independent experiments. Data are presented as the mean ± SE. * *P* < 0.05, Student’s *t*-test.
