# Supplementary material for: Inhibition of Prostaglandin Reductase 2, a Putative Oncogene Overexpressed in Human Pancreatic Adenocarcinoma, Induces Oxidative Stress-Mediated Cell Death Involving xCT and CTH Gene Expressions through 15-Keto-PGE2
Source: PLoS One. 2016 Jan 28;11(1):e0147390. doi: 10.1371/journal.pone.0147390 (PMC4731085; doi:10.1371/journal.pone.0147390)
Supplement: S3 Fig — (DOCX) [file pone.0147390.s005.docx]

**S3 Fig. Silencing of *PTGR2* suppressed expression levels of antioxidative genes xCT and CTH in Capan-2 cells.**

**(A** – **F)** Relative mRNA expression levels of (**A**) PTGR2 (**B**) CTH (**C**) xCT (**D**) GLS1 (**E**) Catalase and (**F**) GSS in si-PTGR2 Capan-2 cells as compared to si-Control cells. Total RNA was harvested and subjected to qPCR analysis and the mRNA levels were normalized to human cyclophilin expression level. mRNA expression levels in si-Control cells were set as 1 and the relative mRNA expression levels in si-PTGR2 cells was presented as values relative to the control. The results are the average of 3 independent experiments each done in triplicate. (**G)** Western blot analysis of the expression levels of xCT, CTH, Catalase and PTGR2 in si-PTGR2 Capan-2 cells. GAPDH served as a loading control. Data are presented as the mean ± SE. * *P* < 0.05, ** *P* < 0.01, Student’s *t*-test.
